# “Leave us alone”: ‘right to the city’ of street vendors along Main North 1 Road, Maseru, Lesotho

**DOI:** 10.1007/s10708-023-10881-y

**Published:** 2023-04-19

**Authors:** Abraham R. Matamanda, Rets’epile C. Kalaoane, James Chakwizira

**Affiliations:** 1 Department of Geography, University of the Free State, Bloemfontein, South Africa; 2 Department of Town and Regional Planning, University of Johannesburg, Johannesburg, South Africa; 3 Department of Urban and Regional Planning, North West University, Potchefstroom, South Africa

**Keywords:** Street vending, Maseru, Urban informality, Permanent temporariness, Right to the city

## Abstract

This article explores the lived daily experiences of street vendors operating along the Main North 1 Road in the CBD of Maseru, Lesotho. This exploration considers how street vendors access and negotiate a claim for the right to the street. The challenges confronting these vendors in their daily hustling, including COVID-19 restrictions, are also examined. A narrative inquiry research design informs this article with data collected from interviews with purposively selected street vendors from Maseru. This primary data was triangulated with document analysis to increase the validity of the findings. The findings highlight strategies employed by vendors in Maseru that include integrating with the formal enterprises, diversifying their trades, resisting and frustrating certain decisions by the local authorities, and contributing to urban blight. A framework for interrogating and understanding street vending and its nuances is postulated based on the findings from Maseru. The article strongly appeals to the authorities to find more benign ways of integrating street vending into the production of cities.

## Introduction

Street vending is a visible informal sector activity in most Global South cities, especially in Africa (Crossa, 2016; Monga et al., 2019). Yet, the contribution of street vending toward urban employment creation and livelihood support is continually overlooked by policymakers and mainstream urban economists in African cities (Young, 2018). This lack of recognition of the value of street vending in the urban formal economy is evident from the blitz targeting street vendors across African countries and the atrocities that authorities imposed upon these street vendors (Musoni, 2010; Young, 2017). A typical example is Operation Sweep Clean, launched in Johannesburg, South Africa, to remove street vendors in downtown Johannesburg and the several blits in Harare, Zimbabwe, that also sought to remove street vendors from the streets (Arias, 2019). Therefore, several authors have debated how street vendors are marginalized in most cities (Adama, 2020; Lata et al., 2019). Street vendors are mostly prohibited from accessing the streets through spatial exclusion nested in the modernist planning approach that views and perceives street vending as an urban nuisance (Popke & Ballard, 2004).

The failure to recognize the utility of street vending in the formal economy has resulted in numerous tensions and conflicts in most African cities between the authorities and the street vendors over issues in licensing, taxation, site of operation sanitation and working conditions (Bandauko & Mandisvika, 2015; Matamanda & Chinozvina, 2020; Resnick, 2020). In response, street vendors employ different strategies to access urban space (Steel, 2012). These strategies by street vendors refer to the right to the city. This right to the urban space refers to the practices of the citizens to define, redefine and shape the use and management of urban spaces (Harvey, 2012; Lefebvre, 1991). This right to the city among the street vendors is evident in their everyday experiences in plying their trade on the street, despite the authorities’ restrictions and regulations.

This study focuses on street vending in Maseru, Lesotho, where street vending is prevalent. The prevalence of street vending in Maseru is no myth and has been escalating (Setšabi & Leduka, 2008). In November 2022, it was estimated that Maseru accommodated about 7 000 shelters for street vendors, of which nearly 5 000 were improperly constructed (The Post, 2022). Considering the socio-economic woes characterized by high unemployment, rising poverty levels, and deteriorating living standards in Maseru, many citizens are left with no option but to engage in street vending (World Bank, 2019). The COVID-19 pandemic has also exacerbated the living conditions among many urbanites who have engaged in street vending (Matamanda et al., 2022). The response of the government towards the street vendors in Maseru is characterized using force, violence, and repression to control the street vendors that are considered a nuisance in the city (Setšabi & Leduka, 2008). For example, in the ruling in the court case “*Baitsokoli and Another v Maseru City Council and Others (CONST/C/1/2004) (CONST/C/1/2004)[2005] LSHC 25 (26 January 2005)*”, the court dismissed the application by the street vendors who had been evicted from Kingsway street in Maseru and claimed the removal jeorpadised their right to life through a denial of their livelihoods based on street vending. Rather, the court insisted that “trading as a street vendor was a deliberate choice and not the only alternative to a living. Thus, the interests and values of livelihood were outweighed.” This case law shows how the rights of street vendors are often repressed and fail to be recognized formally.

Urban geographers have undertaken several studies with a focus on street vending. Issues deliberated include governance of street vending activities (Tucker & Devlin, 2019) and the politics inherent in the daily lives of the vendors (Young, 2017, 2018). Based on the court case cited above, we argue that the everyday experience of street vendors in Maseru is an important site of urban studies and should be taken more seriously by policymakers who often criminalize and repress the sector rather than support the livelihood strategies of street vendors. We also demonstrate that street vendors are not passive actors in creating and recreating urban economic spaces and activities but emerge as innovative and tactful players that employ different strategies to access their rights to the streets where they undertake their enterprises. The everyday experiences of vendors along the MN1R in Maseru are analysed with the question of the right of street vendors to access urban space. The focus is on the resilience and resistance of the individuals engaging in street vending and how they manage to ‘fight’ back against the policies and the formal system restricting their activities.

This study makes two key contributions to urban geography, focusing on street vending in African cities. First, at a theoretical level, it refines the application of the concept of the right to the city through departure from the framework of formal rights instead of seeing rights as claimed and made effective through social struggles that the street vendors engage in. This theoretical contribution also includes understanding the tactical ways vendors employ to access and appropriate urban space, which help them challenge exclusion while eking out a living in a context where they have been entrenched in a state of “permanent temporariness”. Second, the study makes an empirical contribution to the understudied case study, which includes insights into the daily experiences of street vendors in Maseru. Thus, we add to the existing empirical studies on the context-specific nature of urban informality by examining and analyzing these street vendors' lived daily experiences in Maseru. Finally, this work presents an envisioned right-to-the-city modified implementation framework for ‘negotiated and tailored’ vendors access to rights claims and trading spaces in urban areas of developing countries.

### Street vending and right to the city

In his work on the right to the city, Lefebvre (1991) focused on urban space, which he argued was complex and involved different stakeholders with diverse desires and drives that are not reducible to economic imperatives (Lefebvre, 2003). Space is central in the discussions on the right to the city because the contestations and struggles occurring among different stakeholders are mainly on the use and appropriation of urban space. Lefebvre (1991) postulated that space is best conceptualized from three perspectives. First, perceived space refers to the relatively objective and concrete space people encounter daily. Second, conceived space focuses on the mental constructions of space that individuals and groups create. Lastly, lived space is a combination of perceived and conceived space that depicts the individuals’ experience of space in everyday life (Lefebvre, 1991). The right to the city is recognized as a cry and a demand […] a transformed and renewed right to urban life (Lefebvre, 1991: 158).

Rights come with power as individuals or communities determine the norm and whose rights matter. Some proponents (Harvey, 2012; Mitchell, 2003) reasoned that power rests not only with the capitalists who determine the status quo, rather, power can be managed democratically. Nkrumah (1970) illustrated how class structures shape such power in the African context consisting of elites, the proletariat, and the poor, who are often placed at the margins of the social class and have to fight for their rights, which Harvey (2008, 2012) equates to contestations and struggles. Lefebvre (2003) mentions ‘interested parties’ in the revolution for claiming the right to the city. These interested parties include the users of urban space who engage in the appropriation of urban space (Harvey, 2003).

Most importantly, the right to the city is seen as a struggle to “de-alienate” urban space (Purcell, 2014); here, vendors as urban users take control of the street. The citizens’ ability to participate fully in matters that concern them is another dimension of the right to the city where citizens’ participation in the production and management of urban spaces (Harvey, 2012). Purcell (2002) argues that the right to the city is thus about influencing the appropriation of the physical space and determining its use. From their (street vendors), the right to the city is about the use and appropriation of the urban space through accessing the most strategic parts of the street where business is high.

Limited economic and political power among street vendors compromise their ability to influence decisions that concern their trading, making it difficult for them to access specific spaces at times. Resultantly, exploitation may occur as the vendors cannot represent themselves or challenge some decisions that benefit the elites (Adama, 2021). It may be noted that the rights of the vendors are perpetually precarious and never-lasting solutions. This is evident in Kampala, Uganda, where the repression of street vendors followed the country's democratization and political changes (Young, 2017, 2018). They are pushed from one place to the other and never integrated into the formal economy (Kamete, 2018), thus leaving them in the shadows of the formal city in the state of “permanent temporariness” (Yiftachel, 2009).

### Resilience or resistance? The daily struggles of the street vendor

The daily struggle of street vendors includes their criminalization; even when street vending is permitted in certain sites, some skepticism remains about the vendors (Crossa, 2009, 2016). Working spaces for street vendors face differentiated subtle and explicit forms and systems of (non)regulation by municipal and land authorities, including (in)visible vendor informal group/community rules, norms, standards, and practices. Henceforth, when they exist, these markets are contested and nested in political dynamics and clientism regarding the use and appropriation of vending spaces (Huang et al., 2014). For example, the local authority in Kitwe city, Zambia, intended to give a piece of land to some Chinese to build a shopping mall, thus affecting the trading spot for some street vendors in the area (Jongh, 2021). This exclusion and marginalization of vendors is standard practice and is often done, paying little attention to the potential impact and outcomes of such actions on street vendors and their livelihood.

Vendors sometimes lose all their stock which the authorities may impound. For example, the COVID-19 pandemic has resulted in authorities’ immense victimization of street vendors (Coletto et al., 2021). Notwithstanding the need to observe the COVID-19 regulations, some were solely directed at penalizing street vendors for using the pandemic as a scapegoat for such injustices (Kiaka et al., 2021; Matamanda et al., 2022). Street vendors eventually develop strategies to access and negotiate claim rights to the city, as evident in Harare, Zimbabwe, where some have resorted to using different motorized vehicles for trading during the pandemic (Toriro & Chirisa, 2021).

Organized protests and social movements have become a powerful tool that street vendors use (Bénit-Gbaffou, 2016: 5). Collectively, street vendors can influence the appropriation of the streets for vending purposes through formal organizations and associations to fight for their rights (Hummel, 2017). An example has been the advocacy work of the street trader organisations in Johannesburg that negotiated terms of informal traders with the City of Johannesburg following Operation Clean Sweep (Arias, 2019). This somehow legitimizes the vendors and enables their voices to be heard collectively instead of individually as they engage in collective advocacy and agency when deliberating with the authorities (Nyemb, 2017). However, this legal process is not an option for many street vendors considering the cost implications of legal procedures (Matamanda & Chinozvina, 2020). Furthermore, street vendors form relationships with formal owners to help fight against their injustices, while other formal shop owners help them ‘hide’ their products when city authorities arrive and conduct blitz (Forkuor et al., 2017).

In some contexts, street vendors have shifted their mode of working as an adaptive strategy. Some street vendors downscale the products they sell by having few stocks (Roever & Skinner, 2016). This strategy allows the vendors to incur minimum losses in raids from the authorities while also making it possible for them to be mobile and move their wares to other locations where business may be brisk (Malefakis, 2019; Steel, 2012). Forkuor et al. (2017) highlight that some street vendors sell their products when they know that the city authorities are not fully operational during morning hours or lunchtime and evening hours. At times, the street vendors may temporarily relocate to other parts of the city, facing less harassment from the authorities (Young, 2018). In Ghana, street vendors in Accra pose as pedestrians by carrying fewer goods to be seen as pedestrians by city authorities during the raid (Anyimah-Ackah, 2007). Other street vendors pay off local authorities ‘legitimacy or protection fees’ to keep them on the streets, increasing their daily expenses (Adama, 2021; Roever & Skinner, 2016: 8).

## Methodology

### Context of the study area

**Fig. 1:**
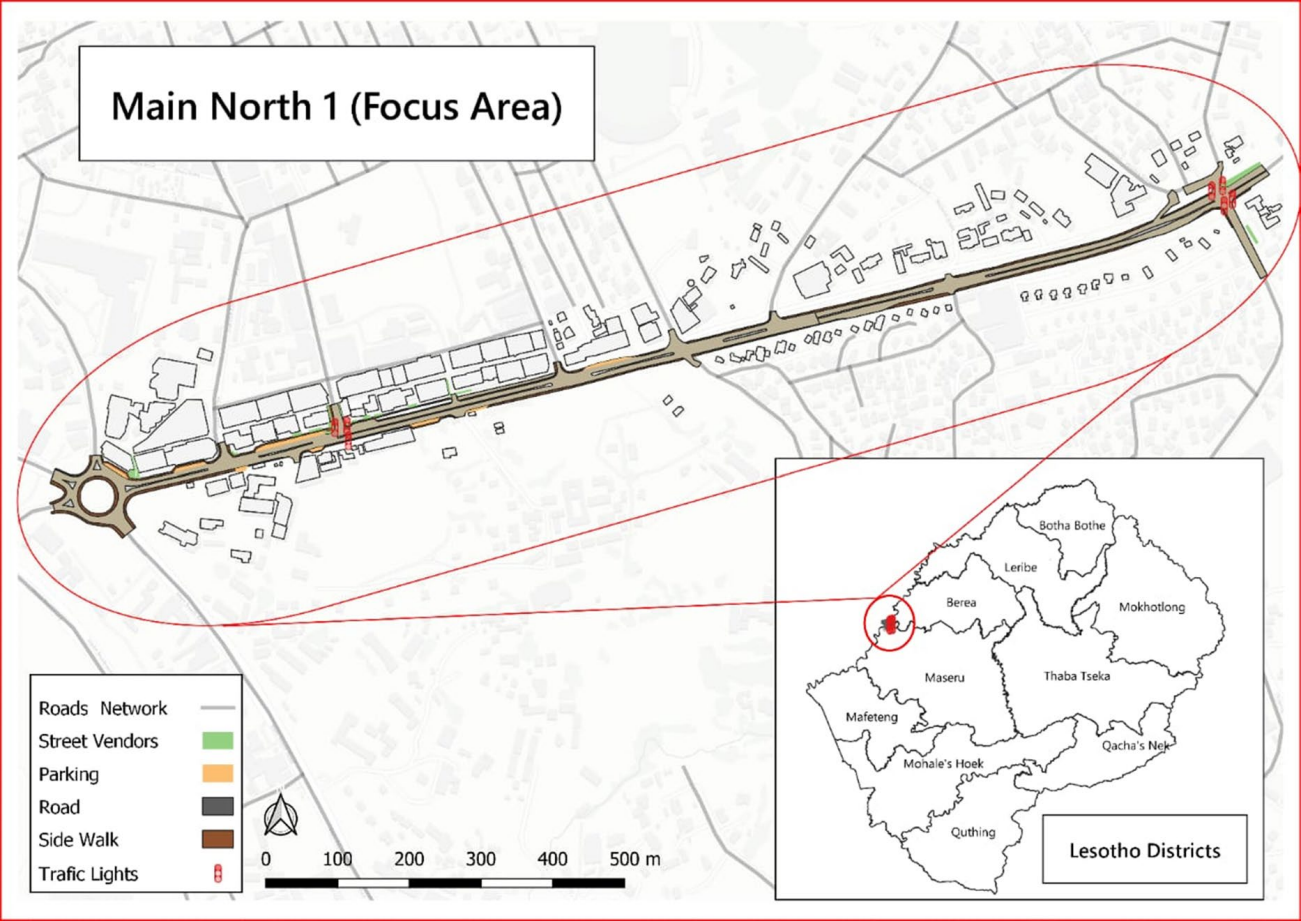
Map showing Mnin North 1 Road, Maseru, Lesotho. *Source*: Authors 2020

Maseru is a colonial city. The central business district (CBD) was designed to accommodate commercial and retail services. The MN1R is the major artery road that cuts through the CBD, making it the most frequently used road. Maseru CBD has a linear development pattern where the businesses are concentrated mainly along the MN1R (see Fig. 1). This makes it the busiest part of the CBD for vehicular and pedestrian movement. Formal planning and regulation in Maseru continue to be guided by the colonial legislation that does not cater to urban informality. Specifically, the Town and Regional Planning Act of 1980 (TRPA), Urban Government Act of 1983 (UGA) and the Market Regulations Act of 1971 (MRA) regulate land use activities in urban Lesotho.

However, these legislations fail to accommodate urban informality. For example, the TRPA does not provide specific guidelines or regulations for urban informality. Rather, city officials uses schedule 1 of the UGA which specifies the duties of the Town Clerk in “Repression and suppression of street vendors in their daily work experience and activities” Section (1) as “to establish, regulate and control markets, to regulate and control trade therein, to let stands or plots in such markets and whenever such markets are established to prohibit, regulate and control trade elsewhere in commodities which are sold at established markets.” This provision in the UGA restricts urban informality activities in urban Lesotho, and the officials thus use this section to criminalise street vendors. Likewise, “Diversified enterprises among the street vendors” Section of the MRA states that “A person who erects any building, tent, booth, shelter or any structure in any town premises without the written permission of the Town Council or District Secretary shall be guilty of an offence and liable on conviction to the penalty prescribed in regulation fifteen.”. This section implies that permission must first be sought from the officials to erect any structure they may use for street vending in Maseru. In “Framework for a sustainable vending access to space and trading rights approach” section, the MRA further states that “No person other than a licenced trader or registered cooperative society shall sell or offer for sale or barter any goods whatever at any place except with the written authority of the Town Council or the District Secretary and subject to any conditions which he may impose.”

### Research design and methods

This qualitative study was guided by a narrative inquiry research design. Narrative inquiry involves collecting data from respondents who narrate their lived experiences and stories (Bell, 2002). We interrogated the street vendors through narrative inquiry and allowed them to reflect on their personal experiences, as postulated by Wang and Geale (2015). In the inquiry, the engagement with the participants also considered the specific location in the storyteller’s landscape that gives meaning to the narrative (Clandinin & Connelly, 2004). This study focused on the physical location where street vendors operate and how the activities in the specific areas affect their experiences. Creswell (2013) recommends small sample sizes for narrative inquiry studies as they enable the researcher to extract deeper meanings from the data collected. Because narrative inquiry research involves collecting data through stories of certain social groups to understand social patterns and get meaning from their views and experiences regarding a particular phenomenon.

We interviewed sixteen street vendors for this study, consistent with Morse (2000), who recommended at least six interviews for narrative inquiry studies. The interview scripts consisted of questions focusing on the type of goods sold, part of the street occupied, challenges experienced in daily experiences and the strategies and activities they employ to claim their right to the street and sustain their enterprises.

Purposive sampling was used to identify the interviewees using semi-structured interviews. These participants were purposively selected from designated areas along the MN1R as depicted in Table 1, showing the demographic profile of the vendors and their respective locations along the MN1R. We also interviewed four shop owners along MN1R using a loose interview guide focusing on the relationship between the street vendors and the shop owners. Observation and photographing was done to capture the environment and other relevant situations relating to street vendors focusing on how they claim their right to the street and the nature of the goods sold. The data was collected between January and February 2021. The positionality of the authors as having engaged in vending in the area previously and contacts still active in the vending business made the research entry and acceptance in conducting the research more accessible.

**Table 1:** The demographic profile of the street vendors and their characteristics

SV	Gender		Goods sold	Types of street vendors			Display type	
	F	M		Fixed stalls	Mobile	Temporary stalls		
1		X	Airtime, cigarettes, sunglasses	X			A shack made out of plastics	
2	X		Vegetables and fruits			X	Mounted on pavement	
3	X		Second hand shoes	X			Shack	
4	X		Fruits and vegetables	X			Shack	
5		X	Second hand shoes and clothes			X	A rail to hang clothes and planks made out of wood to display shoes	
6	X		Maize			X	Use tents as a shelter while cooking maize	
7		X	Frying Russian			X	Use tents as a shelter while frying Russian	
8		X	Fish and bread			X	Use tent as shelter	
9	X		Maize		X		Use tent as shelter to cook maize and travel around the North 1 road selling maize	
10		X	Fruits and vegetables		X		Sell in the middle of the road	
11	X		Fruits and vegetables		X		Sell in the middle of the road	
12		X	Maize and cigarettes			X	Use a tent as a shield while cooking maize and a bagpack to store cigarettes	
13	X		Potato chips and fish			X	Food kiosk	
14	X	X	Second hand clothes		X		Car to display clothes	
15			Second hand clothes and shoes		X		Use own car to display	
16	X		Fruits and vegetables		X		Wheelbarrow	

The researchers were identified as part of the “extended vending family and community” doing research that could assist in (re)solving the vending issues in the area. However, given that the researchers made use of an approved research protocol that also took into account voluntary participation, informed consent, and confidentiality of research data, the fieldwork research process was approached with triangulation of sources to cross-validate observations and findings for increased validity and reliability of findings. In the process, an attempt was made to minimize researcher bias in the interpretation and collection of data. Multiple sources of data collection were critical for this study as it added to the validity of the findings analyzed through thematic and textual analysis.

## Findings and discussion

### Diversified enterprises among the street vendors

Few street vendors sell one type of goods besides those only selling roasted maize and second-hand clothes, most sell fruits. This emerging trend is attributed to the nature of the portable and easily carried around fruits. Selling of fruits was justified by several vendors who pointed out “*selling fruits makes it possible to access claim rights to use and appropriation of the streets as the use of wheelbarrows, carts, or small tables do not take much space, and also allows me to be mobile and evade the authorities*.” The rest of the street vendors sell everything from cigarettes, sunglasses, potato chips, fried Russian, airtime and fish. One street vendor reasoned that *“[…] this strategy to sell a range of goods enables me to maximize my daily profits as I do not rely on a single product*”.

Street vendors selling roasted corn usually use wood to light the fire needed to prepare the corn. The smoke fills the area, irritating pedestrians; however, this is one of the most thriving businesses, as most Basotho people love their maize. Such practices contradict the modernity planning values that seek to promote the aesthetics of the city space (Adama, 2020), especially the CBD, where commercial activities are encouraged. For this reason, the respondents highlighted that the officials repeatedly chase them from the CBD as they are regarded as a nuisance. Yet, the respondents highlighted that because they defy these officials and continue with the maize business, it requires little capital since most people have maize in their fields. As indicated by respondents, this type of business significantly increased along this street after implementing COVID-19 lockdown regulations. One interviewee explained, “*I was working as a cashier on these streets in one of the shops, but when COVID hit, I was one of the people that lost their jobs, so I had to find an alternative that needed as little money as possible.”*

It was evident from the interviews with respondents that reasons for selling along the MN1R included the high volume of pedestrians who made it possible for business viability. An interviewee remarked, " Main North 1 Road is next to bus stops; thus, it is very busy with many people walking and driving past.” It emerges in this regard that bus stops provide lucrative sites for street vendors. This is consistent with the conception by Purcell (2014) of citizens accessing their claim rights and de-alienating spaces where they are restricted to operate from. It also follows that planners often have failed to adequately position some services based on the notion of modernity alluded to by Kamete (2020), which forces the vendors to take matters into their hands and strategically position themselves. Matamanda and Chinozvina (2020) have also highlighted such positioning for Harare, where the local authority establishes trading spaces for street vendors away from the CBD, forcing them to come back ‘illegally’ in the city center where people are.

Again, the respondents all agreed with great enthusiasm that MN1R is a viable space for their business because it is closer to economic activities like the newly built hospital, garage and bars. Moreover, there is a high volume of traffic since the road is used by all residents residing in the northern side of Maseru. This high volume is also attributed to individuals coming from different districts to the city to access different economic activities. This is because most economic activities are centered in Maseru CBD, out of ten districts, the northern part of Maseru is made up of four.

### Repression and suppression of street vendors in their daily work experience and activities

The street vendors indicated that repression from the municipality was the main challenge they grappled with from time to time. This was evident in harassment, bullying from the local police and demolitions of their businesses that happened so often. Street vendor 8, a man who recently moved back home from SA due to unsteady jobs he used to work at, broke down as he said.

Few weeks ago, on the other road of the town, street vendors’ shacks were moved overnight at Ha Thetsane without them knowing, only to wake up in the morning and find nothing. Their whole lives changed […] Although I want to expand my business by making a shelter for my goods, I am afraid once I do that, they will evict me too

The remarks made by this street vendor resonate with the provisions of “Diversified enterprises among the street vendors” section of the MRA that regulates the erection of any structures for street vending. While for street vendors selling outside the premises of big supermarkets, competition is high because most people prefer to buy from supermarkets and fear being evicted by the municipality since they have no rights to the land they are located on. Bad weather was also identified by many as stifling the vendors’ right to the streets. However, that does not stop them from selling even on rainy days, as explained by one respondent:

“If you pass here often, you will see us we don’t stop even when it is raining. We only leave when it is time to knock off. You will see us. You should pass here someday”.

This confirms the challenges facing the street vendors and how vulnerable the sector is as postulated by Kiaka et al. (2021) and Forkuor et al. (2017).

### Effects of COVID-19 on the street vendors

Like any other industry, COVID-19 hit hard on the street vendors, and during interviews, this was the touchiest subject to discuss with the street vendors. The street vendors selling perishable products said they had to throw away their fruits and vegetables because the lockdown took longer than they imagined. Although they felt they were offering essential services like supermarkets, they were ordered to close their businesses throughout the lockdown. Only 3 out of all the 16 interviewed street vendors received a relief fund of about R500 once-off. A respondent narrated the aftermath of COVID-19, saying *“I buy my stock which are shoes from South Africa, so when the borders were closed and still are, it is challenging to keep the business going to an extent where we illegally cross the border to keep the bread on the table”.* The nature of this sector that is not recognized meant they could not receive government grants to sustain their businesses. Moreover, the street vendors supply chains were disrupted during COVID-19, as illustrated by Kiaka et al. (2021), showing how the pandemic was used to penalize the street vendors.

### Access to spaces used by street vendors along Main North 1 Road

However, due to different categories of street vendors selling along this road, the second street vendor indicated that,

For me I didn’t choose it, I was just looking for a place to sell and because I was a mobile seller, I saw this place and decided to put a stall, there were very few of them then.

**Fig. 2:**
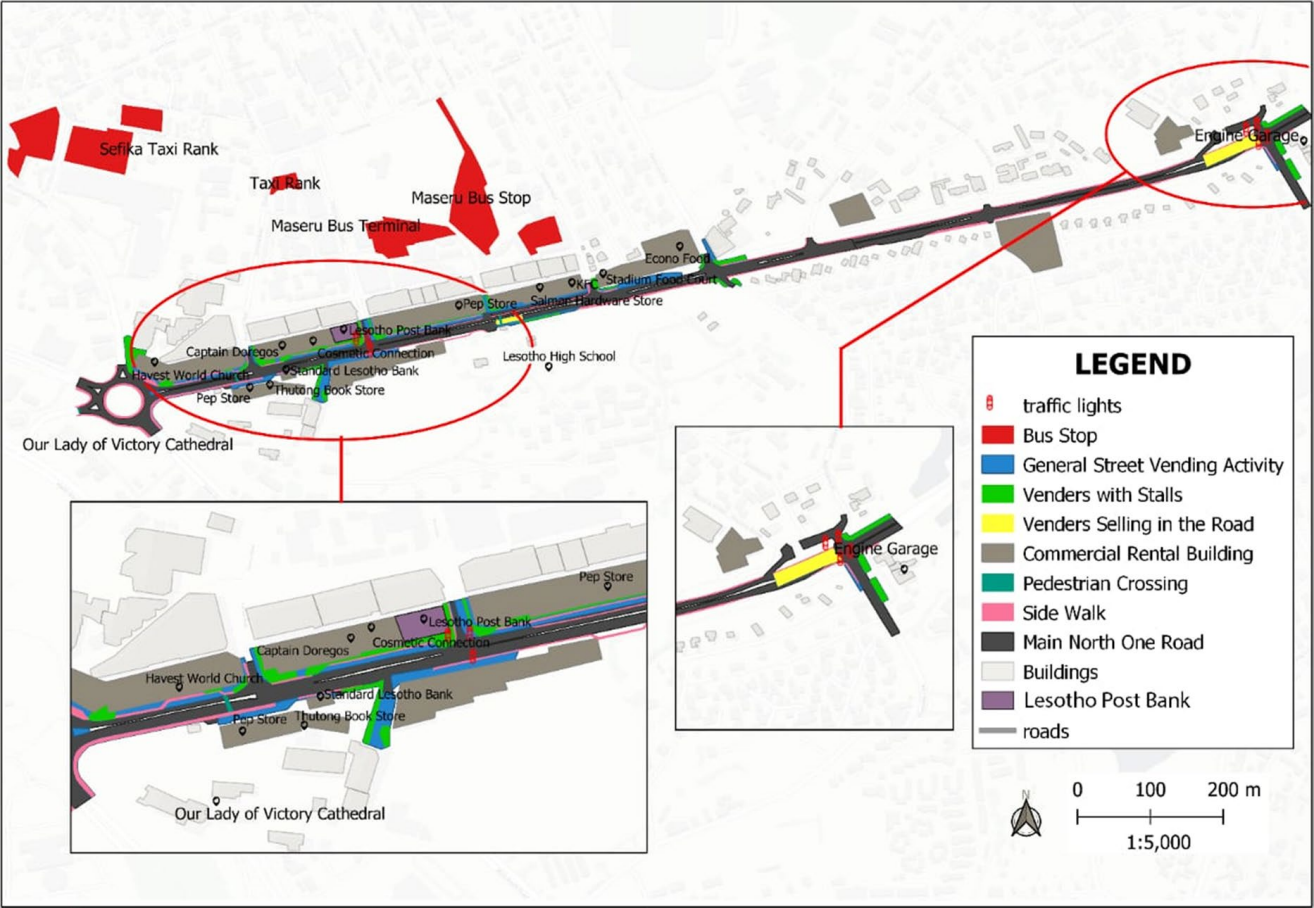
Activities by street vendors along the MN1R. *Source*: Authors 2020

**Fig. 3:**
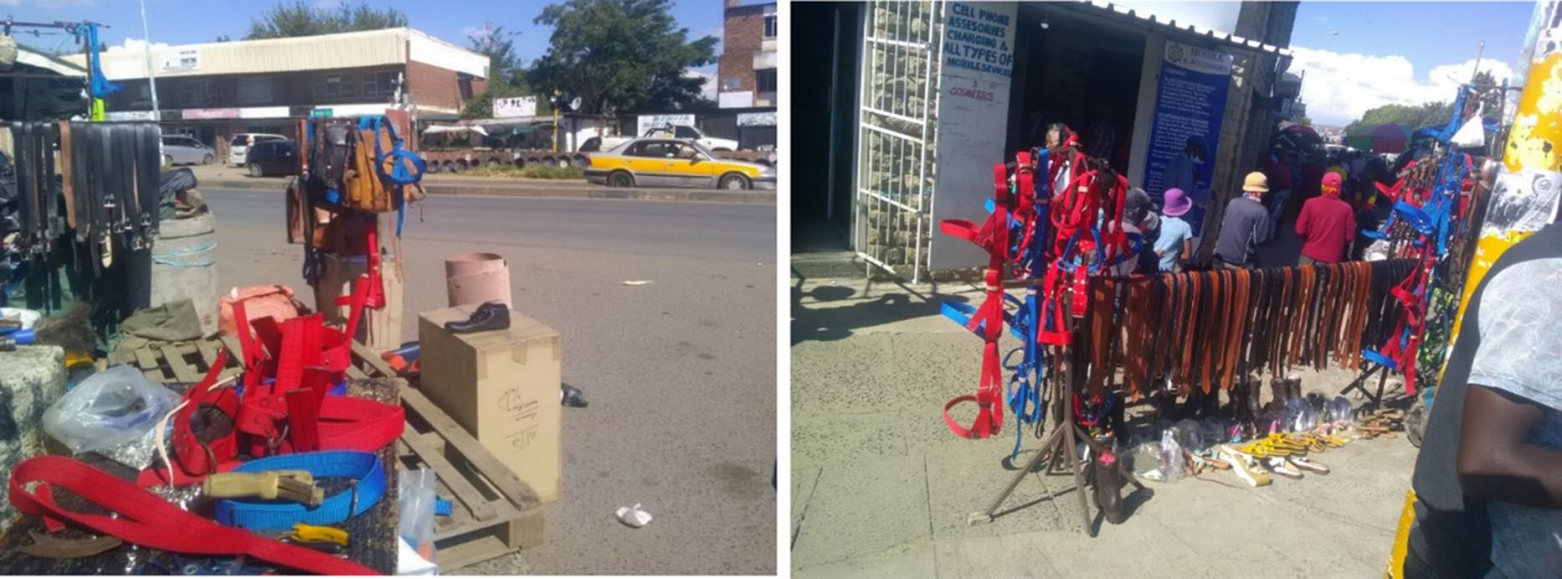
Part of the MN1R used by street vendors to sell goods. *Source*: Authors 2020

Street vendors have also identified some space along the MN1R where they have managed to lay a claim and control (see Fig. 2). Figure 3 shows how some street vendors selling leather products have occupied part of the road meant for parking and used it to display their merchandise. The street vendor has positioned this stand at the intersection of the road from bus stops into the main street, making it hard for cars to see traffic from the left and right sides before entering the MN1R. This claim to the street shows the power exhibited by the street vendors in occupying the street as suggested by Harvey (2008) and Purcell (2014) that those with a stake in the urban space engage in some ‘struggles’ to ‘dealienate’ urban space and assume its control. However, although claiming their right to urban space, the negative effects of some of the informal enterprises come to the fore.

**Fig. 4:**
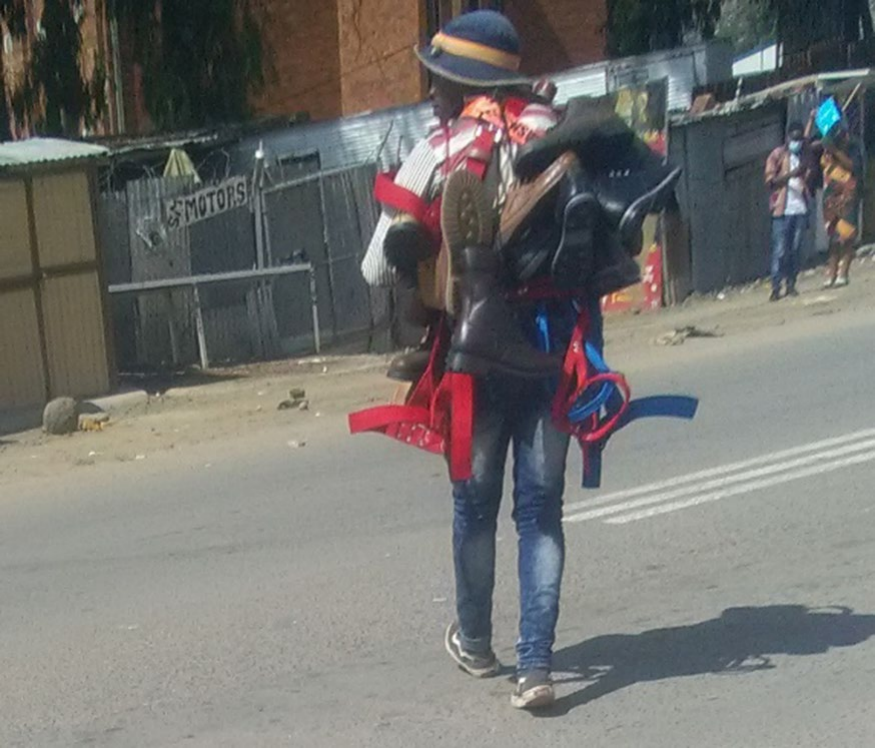
A mobile street vendor selling shoes in the middle of the MN1R. *Source*: Authors 2020

Figure 4 shows a mobile street vendor carrying the second-hand shoes he sells to places with more traffic along the MN1R. This street vendor depicts the street as a flexible space that is not fixed relatively. Vendors use the same strategy in many contexts (Steel, 2012; Malefakis, 2019; Anyimah-Ackah, 2007). One particular street vendor explained that “the street is a complex mosaic that is dynamic and can be accessed in different ways.”

The weather in Maseru can be very extreme, including harsh colds and torrential downpours. It emerged that some street vendors now use their cars as trading spots. One vendor indicated that, “I park my car along the street and then sell my wares from the car”. This is an emerging trend that Toriro and Chirisa (2021) have also highlighted as the mode of operation of street vendors in the COVID-19 era in Harare, Zimbabwe. Such actions affirm the appropriation of the streets as alluded to by Lefebvre (1991) and indicate the state of permanent temporariness explained by Yiftachel (2009) as the vendors operate from such temporary spaces and remain in the shadows of the formal city.

### Relationships with the formal sector

**Fig. 5:**
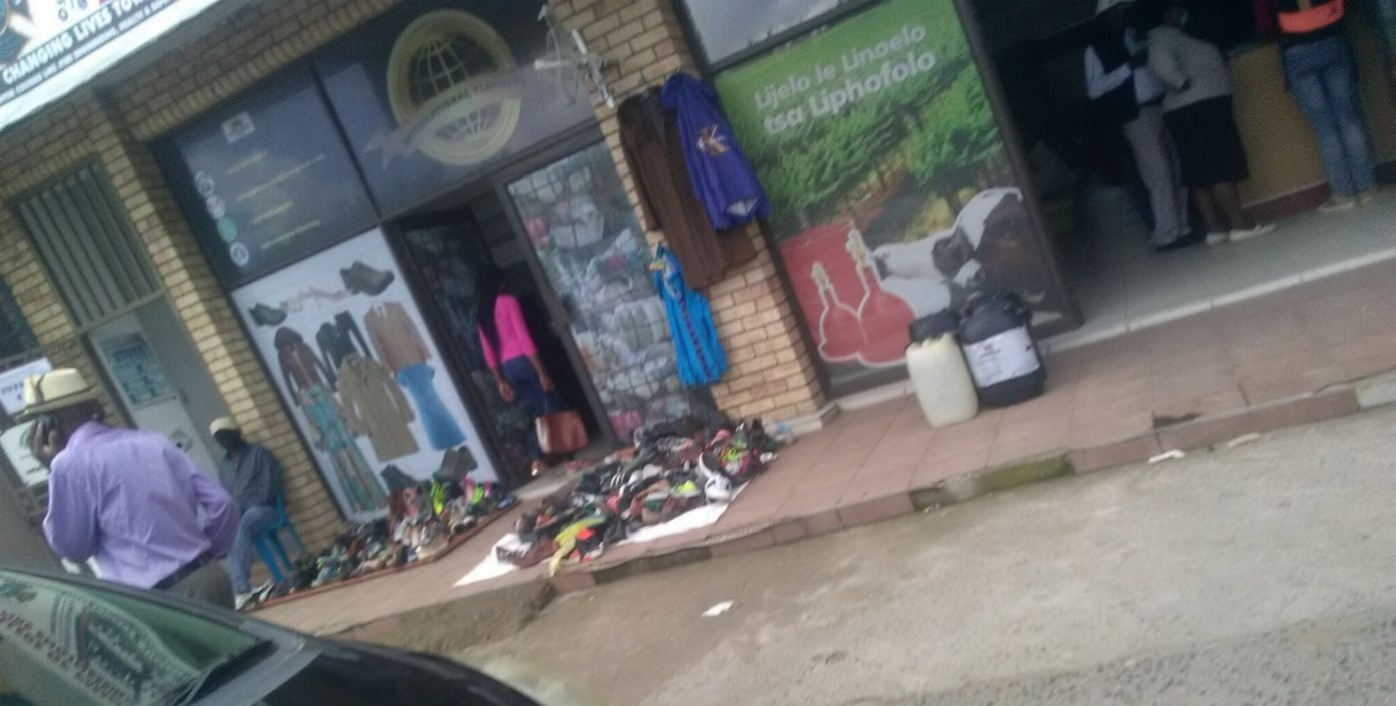
Street vendors located outside formal enterprises selling second-hand clothes. *Source:* Authors 2020

The relationship between the formal and informal sectors has always been perceived as antagonistic (Kamete, 2017). However, the interviews along MN1R highlighted the seemingly symbiotic relationship between these two sectors. They showed how street vendors employ tactical ways to ascertain the use of the urban space by establishing alliances with the formal sector. First, some street vendors operated outside some retail shops selling their ware. Figure 5 shows some second-hand clothes displayed on the walls of the formal shop while some of the merchandise was displayed on the pavement. The shop owner explained that “the street vendors increase their income by providing one-stop shopping.” Popke and Ballard (2004) confirmed this, noting that most street vendors locate outside formal shops and have formed relationships with formal shop owners to sell outside their premises. However, another shop owner lamented that “the street vendors are a nuisance as they block customers from coming in a while also disrupting those who want to do window shopping.”

Second, it was revealed that some street vendors have agreed with some shop owners, especially the Chinese, who allow them to sell in front of their shops. The vendors work for Chinese shops for a commission. Hence, they are allowed to operate in front of these shops. This relationship between the Chinese shop owners and the street vendors is symbiotic. It brings to attention some interesting dynamics relating to vendors eking their livelihoods while the shop owners become their middlemen. One such shop owner pointed out that “we sell our wares to the vendors mostly in large quantities, which the vendors then buy and sell on the street as single units, thus pushing our volumes as the vendors constantly come back to restock.” This informal relationship depicts the forged relations that the vendors employ to remain on the streets, confirming the permanent temporariness of informality articulated by Yiftachel (2009). These street vendors remain entrenched in this cycle of deprivation in accessing the formal, regulated urban space. Although the state can influence the dynamics in the CBD regarding the zoning and land uses, the shop owners in this instance have assumed the power to appropriate the lived space to the street vendors.

**Fig. 6:**
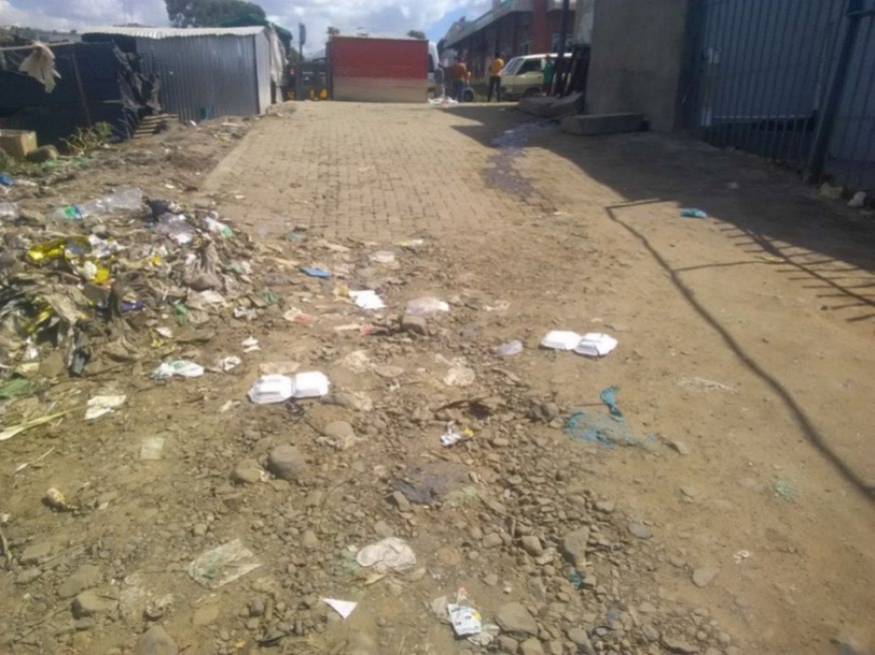
Pavement between a gas station and street vendors used as a dumping site and for public urination along the MN1R. *Source*: Authors 2020

Third, some shops store the wares for street vendors overnight or during the day. This relationship is widely reported in the literature by scholars such as Forkuor et al. (2017). This relationship involving storing the goods by the formal shops is essential to the survival and profitability of the street vendors’ businesses because it lowers the risk of vendors losing their wares in police raids. It allows the vendors to move around with little stock. Once it is finished, they promptly take some more in the formal shops. At times these street vendors who enter into arrangements with the formal shops also get the privilege of accessing staff toilets which proved to be a significant challenge considering the limited availability of public toilets along the MN1R. For most respondents, it was apparent that they use the streets as restrooms owing to the lack of accessible public toilets. This behavior symbolizes another claim these street vendors make about using the street. Although it is unacceptable behavior, they remain with no option but to use alleys as their toilets (see Fig. 6). This has become a norm to male street vendors and does not affect them. A pedestrian asked to comment on such behavior remarked that “*This is Lesotho for you, any man can pee anywhere, and we pass through and do our business. It does not scare us anymore because this is our daily reality.*

However, the lack of friendly, readily available public toilets creates a gender and sanitation challenge, especially for female vendors, as they cannot squat in the same places men use as open defection places. Another concern has been the generated solid waste that the local authority does not collect. Left with no alternative, the street vendors end up littering and dumping their waste illegally, creating an urban malaise and possible threat to public health.

**Fig. 7:**
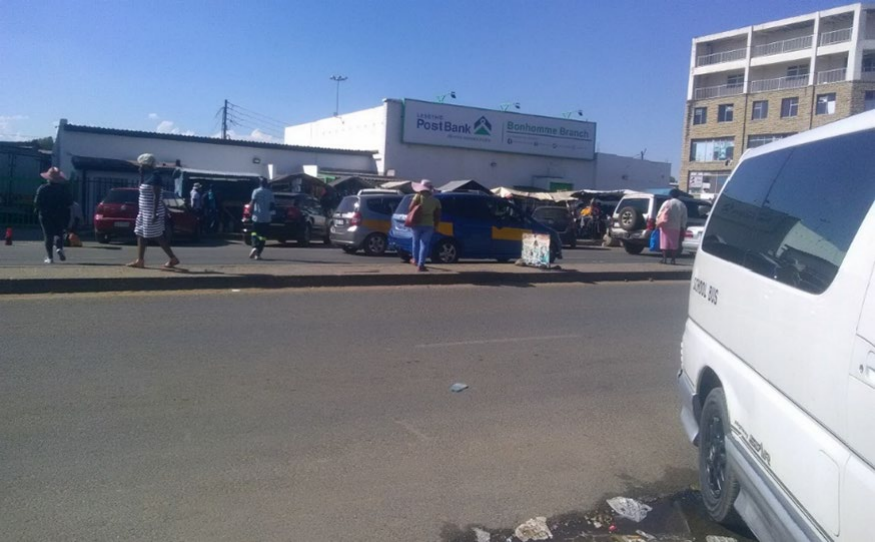
Street vendors surrounding Post Bank. *Source*: Authors 2022

Figure 7 shows a portion of the MN1R with street vendors located in front of the Post Bank. This case highlights how street vendors engage in resistance, highlighted by Lefebvre (1991) and Harvey (2012). The vendors argued that the bank was built years after they started their business; hence, they were entitled to the space. During its foundation phase, the municipality tried to negotiate with street vendors, it seemed for a while that most street vendors relocated. Still, after the building started to operate, both the old and new came back. A respondent commented:

[…], the municipality has a problem with us, so they can come back any time of their choosing and decide to vacate us without any notice. I am working here and here is the Post Bank behind me; when this was constructed, they told us that we should shift our stalls to allow for construction, and in return, we were promised that they would build nice stalls for everyone. We have been waiting ever since, and nothing has happened.

The claim for access to space is evident here, and the situation depicts the vendors' struggles in resisting being moved to an alternative area. Such observations confirm the argument by Harvey (2008), who indicated that citizens claim access to use and ownership of certain parts of the city. Regardless of the formal entitlement that the bank has to the space, the vendors have remained adamant on the premise that they were supposed to be allocated the place for their small enterprises. Although these vendors may continue to claim their right to this space, they have been placed in what Yiftachel (2009) terms ‘permanent temporariness” as they do not have the ability and capacity to occupy the space formally but will continue trading informally unless the local authority allocates a formal space for them that is closer to the people.

### “Leave us alone”

When asked what they would like the local authority to do to help grow their businesses and enable them to enjoy their right to the city through engagement in street vending operations, eleven street vendors wanted nothing from the municipality except to be left alone. A respondent explained that “the authorities must **leave us alone**. They have shown they do not care about us, and we must find our way against the challenges we experience.” Three opined that the municipality should grant them some form of tenure to allow them to operate at designated spaces along the MN1R, and one respondent supported this.

We need legal documents that will allow us to be here because we know the rules, mount our shacks away from the road, and make sure we do not obstruct traffic. We have an excellent plan. Also, we try to clean the place, so the city is not dirty. However, we are prepared to keep on moving up and down until they understand we need to earn our living and should not be stopped because there are no jobs in this country.

The remaining two wanted shelter to use during heavy storms and rain. In that way, they can easily protect their fresh produce. The overarching response in this regard was that the street vendors wanted limited interference from the city officials in their daily duties. This reflects the street vendors' desire to be left alone, even though some indicated they would be happy if the local authority would erect structures for them.

## Framework for a sustainable vending access to space and trading rights approach

**Fig. 8:**
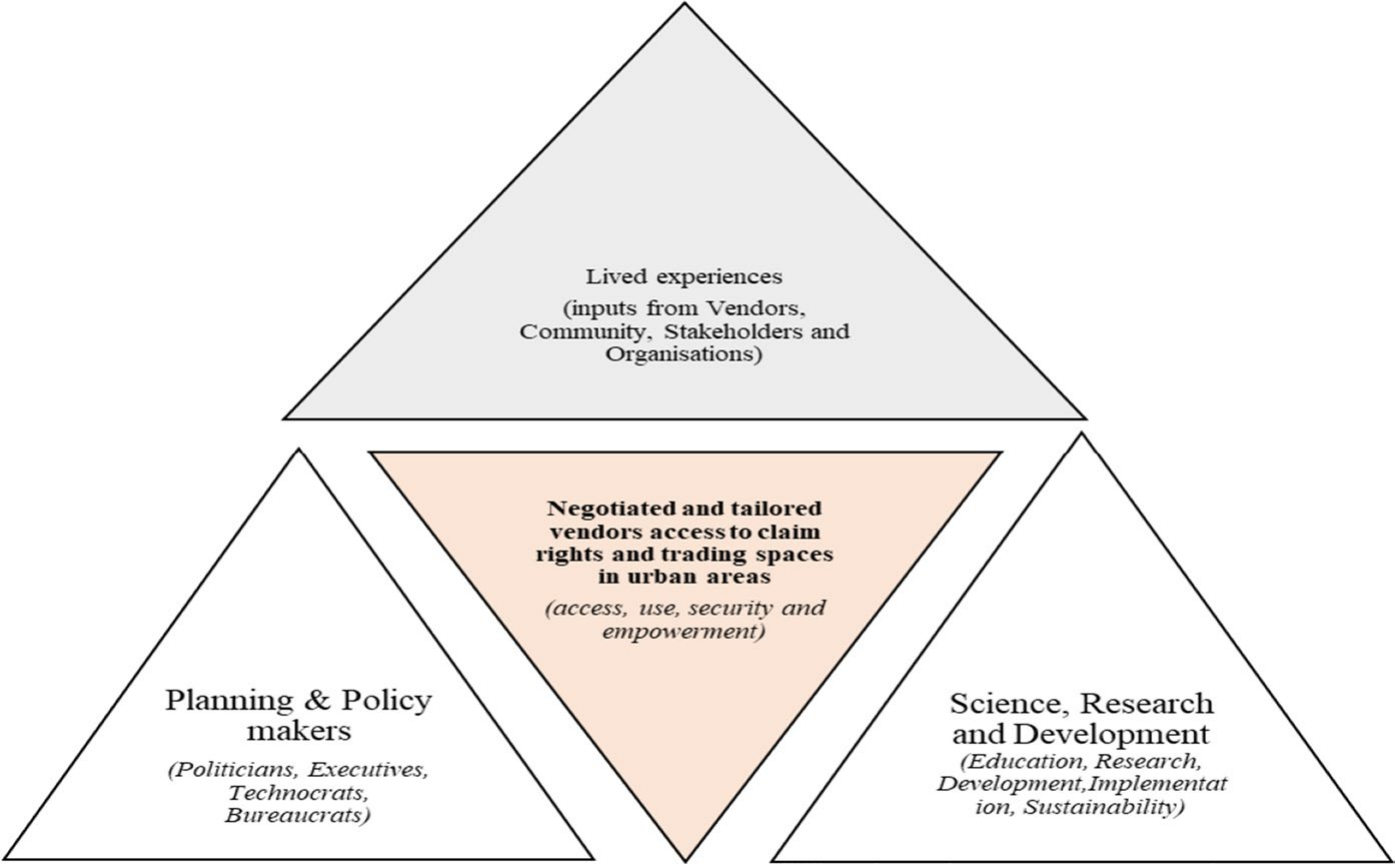
Framework for negotiated and tailored vendors access to rights claims and trading spaces in urban areas. *Source*: Authors creation 2022

Considering the clear narrative of “leave us alone” but being mindful of the reality that land and municipal governance systems cannot turn a blind eye to any development happening in their areas of jurisdiction, vending, included, Fig. 8., was generated as a sequel and logical conclusion to the discussion. The framework was developed based on the empirical findings generated from Maseru, formal legislation guiding use and access of urban space in the case study area and insights from the authors' experience of interacting with and research conducted with street vendors. The framework for negotiated and tailored vendors' access to rights claims and trading spaces in cities triad locates that the conversation revolves around access or lack of access to trading (economic) spaces. Also the (in)ability to use apportioned or claimed or identified economic vending nodes/sites for economic opportunity restricts street vendors’ rights in cities. Moreover, street vendors activities are compromised by the security or lack of insecurity in engaging in the vending and trading enterprise as both a business and enterprise given the (Il)legality and land use and zoning schemes/codes provisions as well as the impact of the intersections of these linked and interrelated factors. Overall, these factors advance a spatial environment for empowering or disempowering persons involved in the (re)negotiated and (re)tailored vendors access to rights claims and trading spaces in urban areas. The outcomes of these intersectionalities of power and politics is a site for spatial (in)equalities and (in)justice and (un)sustainable development planning and management approaches and or tactical or tactless urbanism in attempts to curate the street vendors' access to rights claims and trading in urban spaces.

Several interventions to activate the implementation of the framework for negotiated and tailored vendors access to rights claims and trading spaces in urban areas (Fig. 8), can be organized themed and clustered in the multi-governance system of networks, collaborators, and partners. A sustainable framework for negotiated and tailored vendors access to rights claims and trading spaces in urban areas triad has to be supported by a refreshed and alternative model. The key pillars of the triad were developed as a graphical synthesis and representation of the following processes and methodology:

- Analysis of existing lived experiences and constraints for and by vendors,
- Critical review and gap analysis of legislative and policy framework in support or against street vending,
- Gap analysis and review of street vending choice, location and physical siting of trading areas, type and model structure of vending structures and architectural design, layout and display tables forms and types as well as vending structure construction materials used e.g. wood, plastics, polythene etc.
- A problem tree analysis method and author’s brainstorming session on the vending issues and challenges in Maseru was conducted. This exrcise helped in shaping and refining the development of the sustainable framework for negotiated and tailored vendors access to rights claims and trading spaces in urban areas triad as presented in Fig. 8.

The outcome of these four (4) above mentioned procedural analytical stages, culminated in the development of the negotiated and tailored vendors access to claim rights and trading spaces in urban areas triad as represented graphically in Fig. 8. Generally, in the triad the three (3) key aspects, namely: lived experiences, planning and policy makers and science, research and development are inter-related, inter-dependent and complementary to each other. They are arranged in a state of dynamic equilibrium in which changes in one triad trigger a chain reaction in response from the other triad aspects, until the vending dynamic equilibrium is restored or approached. In the model, planning and policymakers (i.e. politicians, executives, technocrats and bureaucrats) have to (re)negotiate and co-produce the provision, management and sanitatisaion of urban spaces. These policymakers ought to collaborate and partner with the vendors and must have a clear sense and understanding of access to rights claims and trading spaces in urban areas and how this can be infused within the legal and policy frameworks of the respective urban areas. This is especially critical considering the perceptions of the authorities towards street vending, which is not prioritized as a livelihood strategy for the majority of the urban poor. Although having some flaws, lessons can be drawn from the engagements between trader organisations and Johannesburg City Council following Operation Clean Sweep enactment in the city. One of the positive outcomes of Operation Clean Sweep in Johannesburg was how the activity and programme paved the way for mutual and collaborative engagements between the street vendors, policymakers and various social movements.

This has implications in terms of the (re)spatial clustering, dispersal, targeting and branding of negotiated and tailored vendors access to rights claims and trading spaces in urban areas that have happened before. Science, research and development also has a critical role in further developing and improving the framework for negotiated and tailored vendors access to rights claims and trading spaces in cities. The role of science is related to the formal legislation regulating land uses in Maseru which has no place for urban informality mainly due to the Eurocentric nature of the formal laws that criminalise urban informality. In addition, science can play a role in the development of simple, robust and appropriate mobile vending structures (e.g. collapsible, foldable, adaptable designs and structures etc.) that meet minimum building/construction and World Health Organisation (WHO) operation standards for water and sanitation hygiene requirements as an example. Therefore, the need for (re)curriculum and updating of planning education and ideologies which ‘shape’ planners so that they can incorporate better the other viewpoints of vending and trading in training as part of decolonizing western orientated biased curriculum. Such a process will help to provide expression and space for post-colony identities of vendors access to rights claims and trading spaces in urban areas of Africa as typified by the case in Maseru, Lesotho.

The need to harness the power of big-data and analytics and robotics and even drones in data gathering and intelligence development of systems, portable and mobile multipurpose trading spaces is an area ripe for further development as the world of work changes moving into the next century as an example. Indeed, the framework for negotiated and tailored vendors access to rights claims and trading spaces in urban areas success of the triad is anchored on a better and improved understanding and processing of the lived experiences (vendors, community members, stakeholders and organisations). This understanding enables total and complete solutions that are inclusive and sensitive to all aspects linked and connected to the framework for negotiated and tailored vendors access to rights claims and trading spaces in cities. Through these negotiations, value chain and dividends can be achieved so that when the “vendors” speak of “leave us alone” metaphor, this is not seen as urban rights metaphors and empty signifies for politicking. Rather, is a practical solution acceptable to all as part of furthering the inclusive city agenda.

The need for mindshits and sets renewal and updating by all actors is critical. Areas that can be developed for better integration and development could include the following: Issuing of ‘special staff cards’ for vendors to access toilets of operating precinct/zone vending station radius, zonal vending trading engagement champions, business/vendors Integrated development committees and area based vending forums. Through the establishment of these (in)formal engagement platforms and mechanism, ways for changing and re-inventing the perception of vendors from “leave us alone” and the authorities from seeing them as “an urban nuisance” can be attempted. Policy and planning engagement modes would follow suit and a new vending geographyscape can emerge and be imagined. Overall, the sustainable framework for negotiated and tailored vendors access to rights claims and trading spaces in urban areas triad model strength and foundation lies in sustainability. The collaborative and partnership development model that underpins the framework attests to the centrality of sustainability. This under-girding approach provides the golden invisible thread that ties together the sustainable framework for negotiated and tailored vendors access to rights claims and trading spaces in urban areas triad. Without the active and meaningful participation and engagement of all actors and role players that comprise the triad, the sustainability and success of the framework is threatened. However, with the active and meaningful participation and engagement of all actors and role players that comprise the triad activated and harnesses, the sustainability and impact of the triad is advanced.

## Conclusion

Three key observations are made in this article relating to street vendors and their claim to access right to vending space in the city. First, the study confirmed that street vending is an intricate part of Maseru's production of urban space, as reported for other African cities such as Abuja, Harare, and Johannesburg. This intricacy is evident in how street vendors claim and control certain parts of the street, including parking areas, pavements, the middle of the road, and even in front of formal shops. Second, Maseru has elements of resistance and resilience associated with street vending. Unlike other street vendors’ violent protests and resistance, the Maseru case shows some subtle resistance with minor physical and violent clashes between the local authorities and the street vendors.

In claiming their rights, street vendors have also devised mechanisms that include selling and operating from their cars. This strategy is also largely attributed to the COVID-19 restrictions that have prompted the vendors to sell from their vehicles. Third, the symbiotic relationship between the street vendors and some formal enterprises was shown. This highlights the integration of the two sectors, as Kamete (2020) alluded to. Although most proponents have highlighted the relationship between the formal and informal in terms of storing goods, the Maseru case shows the signs of a “partnership” that includes the distribution chain of the goods. This distribution chain consists of the street vendor who sells the small items to the pedestrians, saving them the hustle of getting into the shops when they only need to buy a single thing. Moreover, some shop owners give access to the street vendors to their staff toilets, which helps minimize environmental pollution. The alternative for most is using the alleys as toilets.

Finally, the street vendors’ right to accessing vending space along the MN1R is riddled with various challenges beyond the local authorities. Recently, the COVID-19 pandemic has also come to threaten the right of the vendors through lockdown restrictions and social distancing, which has prompted some vendors to operate from their vehicles. It has emerged that although the need for street vendors to claim their right to vending space in the city, they tend to infringe on other stakeholders' rights. First, urban decay is becoming a problem due to the lack of environmental stewardship among some vendors who do not consider the rights of the environment and public space by dumping waste on undesignated places and using alleys as public toilets. Second, there is a blurred line between the formal and informal as some shop owners complained of the implications of the street vendors on their businesses.

Here, there are two sides to the coin, those shop owners who have forged some partnerships with the street vendors who act as their agencies and benefit from the street vendors. In contrast, those who have not entered such agreements with the street vendors complained that their businesses were affected negatively due to the competition from the vendors. The sight of vendors in certain places, such as in front of the Post Bank is not good as people do not feel safe walking into and out of a bank whose entrance is crowded by street vendors. Such security concerns jeopardize the bank's operations, showing how the vendors also trample on the rights of the formal institution in claiming their right to the space previously promised to them by the local authority. This points to the need by the local authority in regulating street vending and finding appropriate market spaces for them instead of leaving the vendors in a state of ‘permanent temporariness’ yet compromising the effective operation of the formal enterprises.

Any antagonistic relationship between the street vendors and the authorities is not favorable ground for meaningful engagement towards regularizing street vendors. The sentiment by the vendors to be left alone may largely be attributed to the authorities’ activities and programs seeking to modernize the city and do away with the vendors. Also, the situation at Post Bank confirms the manners in which the authorities least prioritize the vendors and consider the higher returns from the formal institutions rather than establishing a vending market. Based on this study, further research may be undertaken in Maseru to examine the contribution of street vending to GDP and the risks of the informal sector on the sustained development and growth of the CBD. Further research may also be undertaken building on the framework for a sustainable vending access to space and trading rights approach. This study may be a comparative case study such that it investigates the key theoretical underpinnings emanating from this study. Additionally, granted that the study focus did not interrogate issues on vendor associations and non-governmental organisations (NGOs) and street vending space and power dynamics, we however recognize these as areas for future research.
